# Acknowledgement to Reviewers of *Polymers* in 2018

**DOI:** 10.3390/polym11010150

**Published:** 2019-01-16

**Authors:** Polymers Editorial Office

**Affiliations:** MDPI, St. Alban-Anlage 66, 4052 Basel, Switzerland

Rigorous peer-review is the corner-stone of high-quality academic publishing. The editorial team greatly appreciates the reviewers who contributed their knowledge and expertise to the journal’s editorial process over the past 12 months. In 2018, a total of 1424 papers were published in the journal, with a median time to first decision of 14 days and a median time to publication of 33 days. The editors would like to express their sincere gratitude to the following reviewers for their cooperation and dedication in 2018:

Abbes, BoussadLiu, YangyangAbdollahzadeh Jamalabadi, Mohammad YaghoubLiu, Chien-HaoAbel, Steven M.Liu, ZhitianAbou Neel, Ensanya A.Liu, Shi-XiaAbuin, GracielaLiu, Ying-LingAchilias, Dimitris S.Liu, SongpoAckar, ĐurđicaLiu, BoAgeitos, Jose ManuelLiu, XifengAgliari, ElenaLiu, TianyuAgrawal, Kumar VaroonLiu, MingluAgrawal, RichaLiu, PengfeiAhlenstiel, ChantelleLiu, XuqingAhmad, ZeeshanLiu, YaodongAhmed, MaryaLiu, BaiquanAkiba, IsamuLiu, Cheuk LunAkitsu, TetsuyaLiu, XiaoboAla, GuidoLiu, YanbiaoAlabastri, AlessandroLiu, MingxianAlam, Mohammad ParvezLiu, XianjieAl-Bataineh, SameerLiu, MinghuaAlbo, JonathanLiu, LibinAlcock, BenLiu, XianhuAlcoutlabi, MatazLiu, ChuanjunAlegre, CinthiaLiu, Qing QuanAlexander, CameronLiu, TianboAli Ahmadi, MohammadLiverani, LilianaAlmeida, HenriqueLizundia, ErlantzAlongi, JennyLo Giudice, GiuseppeAlt, HelmutLoh, Xian JunAltarawneh, MohammednoorLolla, DineshAltstädt, VolkerLongoni, GianlucaÁlvarez, Carlos Manuel NegroLopes, Carla M.Alvarez-Alonso, PabloLopez, FrancescoAmarowicz, RyszardLópez De Lacalle, Luis NorbertoAmer, HassanLópez-Garzón, F.J.Ammu, PrhashannaLópez-Lorente, Ángela InmaculadaAn, SeongpilLoppinet, BenoitAnanikov, Valentine P.Lorenz, AlexanderAncuţa Elena, TiucLotti, NadiaAndreopoulou, Aikaterini K.Lou, TiejiongAndritsch, ThomasLou, SongAndrzejewska, EwaLow, NicholasAngelova, AngelinaLu, YaoAngione, Maria DanielaLu, ShenzhouAnnamalai, Pratheep KumarLu, FachuangAnsaloni, LucaLu, ZhongAntonino, FamulariLu, HongxuAntunes, MarceloLu, Qing-HuaAnyszka, RafałLubczak, JacekAoki, Ken’ichiLuda, Maria PaolaAoyagi, TakeshiŁukasik, RafałApetrei, ConstantinLuna, CarlosAphale, AshishLungu, ClaudiuAppelhans, DietmarLuo, RongcongArad, ShoshanaLuo, XiaolinArao, YoshihikoLuo, YunjunArdelean, Lavinia CosminaLuo, YanhuaArencón, DavidLuo, ShuaiArgüelles-Monal, WaldoLuscombe, ChristineAriga, KatsuhikoLusi, MatteoArjmand, MohammadLuxenhofer, RobertArkas, MichaelLuzi, FrancescaArmentano, IlariaLvov, YuriArrabal, PilarŁyszczek, RenataArrieta, MarinaLyu, HailongArrigo, RossellaLyulin, Alexey V.Asai, DaisukeMa, TianyiAshley, JonMadden, DavidAshraf, WardaMadurga, SergioAta, SeisukeMaeda, TomokiAtabaev, TimurMaeda, ShingoAtanase, LeonardMagalhães, Fernão D.Athanasopoulos, NikolaosMagkoev, TamerlanAtrei, AndreaMahata, Manoj KumarAtsawasuwan, PhimonMahmoodi, MehdiAttila, BaloghMaik, FeldmannAttri, PankajMaisch, TimAuclair, NicolasMajewski, LeszekAuriemma, FiniziaMajor, IanAvérous, LucMalakooti, Mohammad HAyesta, IgorMałgorzata, MatusiakAzoug, AurelieMalka, DrorBaba, KoumeiMallavia, RicardoBácskay, IldikóMalucelli, GiulioBadea, IldikoMamane, VictorBadia, Jose D.Mamiński, MariuszBae, Ki HyunMandal, AjayBaggiani, ClaudioManfredi, LuigiBahk, Je-HyeongMangialardi, TeresaBai, JiamingManian, Avinash P.Bai, MingfengManjunath, ManuboluBai, ShiqiangMannell, HannaBai, ShiManos, George C.Baig, ChunggiMansoori, G. AliBaino, FrancescoMao, LeiBaker, PaulMao, ChuanbinBalamurugan, Sreelatha S.Marchesan, SilviaBallard, NicholasMarcinko, Joseph J.Baltriukiene, DaivaMarco, Iolanda DeBanach, MarcinMarega, CarlaBanerjee, Shib ShankarMaric, MilanBao, ChunhuiMarie, EmmanuelleBarandiaran, IratiMarkondeya Raj, PulugurthaBarany, TamasMarpu, Sreekar BabuBarba, Francisco J.Marques, CarlosBarczewski, MateuszMarrocchi, AssuntaBardet, MichelMartella, DanieleBarile, ClaudiaMartinelli, AnnaBarille, RegisMartinez, Virginia MartinezBarmatov, EvgenyMartinho, José GasparBarretta, RaffaeleMartinka, JozefBarry, CarolMartín-Martínez, Francisco J.Barszczewska-Rybarek, IzabelaMartín-Ramos, PabloBarton, David C.Martins, AlbinoBasso, Maria CeciliaMartins Amaro, AnaBastarrachea, Luis J.Martone, AlfonsoBastola, EbinMasaharu, NakayamaBattista, EdmondoMascia, LenoBaudis, StefanMasek, AnnaBauer, FrancoisMassa, SamBauer, EikeMassoud, SalahBayer, IlkerMastrangeli, MassimoBazylińska, UrszulaMatczak-Jon, EwaBeall, GaryMateo, ReyesBear, JosephMatko, VojkoBeard, James D.Matsui, JunBedini, EmilianoMatsukawa, ShingoBednarz, SzczepanMatsumoto, Ken’ichiroBehnood, AliMatsumura, KazuakiBehzadi, ShahedMattinen, Maija-LiisaBella, FedericoMatwijczuk, ArkadiuszBellantone, VincenzoMauriello, GianluigiBellayer, SéverineMauriello, FrancescoBellich, BarbaraMautner, AndreasBellini, CostanzoMawhinney, ThomasBenali, SamiraMayer, ChristianBencardino, FrancescoMcClung, AmberBeneš, HynekMcDaniel, JonathanBeniah, GoliathMcGrattan, Kevin B.Benito, Juan M.McHugh, KevinBenmokrane, BrahimMedellín-Rodríguez, Francisco J.Bento, Rita Nogueira LeiteMederic, PascalBenyahia, BrahimMederski, Piotr S.Bernath, AlexanderMedronho, BrunoBernechea, MaríaMees, MaartenBertani, RobertaMehrali, MehdiBertoldo, MonicaMeier, UrsBertoncello, PaoloMeijide, FranciscoBeuermann, SabineMeille, ValerieBhattacharjya, DhrubajyotiMekonnen, Tizazu H.Bhattacharya, SupriyoMen, YongjunBiałek, MarzenaMeng, FanbenBian, PeiMeng, FanbinBielawski, Christopher W.Meng, HaoBielinski, DariuszMengistie, Desalegn AlemuBieliński, Dariusz M.Mensah, Enoch A.Biernasiuk, AnnaMéo, StéphaneBiernat, JanMessina, FrancescoBikiaris, DimitriosMiao, TianxinBisoyi, Hari KrishnaMichels, JulienBiswas, ShaurjoMichl, Thomas D.Biswas, AniketMickelson, Alan R.Blanco, IgnazioMiculescu, FlorinBoccia, Antonella CaterinaMicutz, MarinBocz, KatalinMiethlinger, JürgenBokias, GeorgiosMignon, ArnBoldt, RegineMiguel, Sónia P.Bolotov, LeonidMija, AliceBolton, KimMilyukov, Vasili A.Bon, VolodymyrMinakshi, ManickamBoonkaew, BenjawanMinato, Ken-ichiroBoota, MuhammadMiquelard-Garnier, GuillaumeBora, MeghaliMisiakos, KonstantinosBorges, João PauloMistretta, Maria ChiaraBorkowski, AndrzejMitov, MichelBormashenko, EdwardMitra, SubhasishBortot, ElianaMitsuhiro, TerakawaBos, Philip J.Mitsumata, TetsuBouquillon, SandrineMitus, AntoniBouropoulos, NikolaosMiura, YoshikoBoy, RamizMiyazaki, ToshikiBoyd, DarrylMizera, AlesBracciale, Maria PaolaMizielińska, MałgorzataBrand, Helmut RMizsei, JánosBranda, FrancescoMizusaki, MasanobuBrás, Ana RitaMo Jeong, HyungBraunecker, WadeMohammed, AlyaaBrebu, MihaiMohammed-Saeid, WaleedBreitenfeld Granadeiro, LuizaMohanta, AntaryamiBrettmann, BlairMohd-Yasin, FaisalBreveglieri, MatteoMolina, MariaBronnikov, SergeiMollica, F.Brosse, NicolasMoncho Jorda, ArturoBrostow, WitoldMondal, KunalBruder, Friedrich KarMonroe, Mary Beth BrowningBrunetto, Priscilla S.Monteil-Rivera, FannyBrunner, AndreasMonti, MarcoBrychcy-Rajska, EwaMoon, Hong ChulBubnov, AlexejMoon, Doo KyungBuchowicz, WłodzimierzMoratti, SteveBucio, EmilioMorávková, ZuzanaBulgariu, LauraMoretti, GiacomoBuller, JensMorgan, AlexanderBumgardner, Joel D.Morganti, PierfrancescoBurkhardt, TheodoreMori, WasukeBurrows, HughMorikawa, AtsushiCabanelas, Juan CarlosMorimura, ShigeruCabral, JaydeeMorisaki, YasuhiroCabrales, LuisMorreale, MarcoCabrera, HumbertoMorris, GordonCabulis, UgisMosnacek, JaroslavCaggiano, AntonioMotta, AntonellaCai, FengMrówczyński, RadosławCai, ChaoMróz, WaldemarCaillol, SylvainMrukiewicz, MateuszCalabrò, VincenzaMu, QingxinCaldera, FabrizioMucha, MariaCalderón, VerónicaMueller, AnjaCametti, CesareMüller, Alejandro J.Camões, AiresMunaò, GianmarcoCampardelli, RobertaMünch, AlexanderCamposeo, AndreaMuñoz, AsunciónCanal, José MariaMunoz Guerra, SebastianCancelliere, PiergiacomoMunteanu, Bogdanel SilvestruCao, YuheMurakami, NaoyaCao, ZhenMurshid, NimerCao, MaoshengMurugan, N. ArulCao, Gui-pingMusmarra, DinoCao, BingMuthukumar, ThangaveluCao, JianyunMuziol, Tadeusz MikolajCarapeto, AnaMystkowska, JoannaCarbone, GiuseppeNaderi Kalali, EhsanCarbone, AlessandraNadolny, ZbigniewCardinaels, RuthNagase, YuCarlos, TéllezNagy, EndreCarneiro, Olga SousaNah, Jae-WoonCarosio, FedericoNair, DevathaCarradori, SimoneNair, Jijeesh RaviCarraro, MassimoNair, Sandeep SudhakaranCarrasco, SergioNair, RemyaCarretero-Gonzalez, JavierNakata, NorioCarsí, MartaNakayama, YuushouCarvalho, LidiaNakazawa, YasumotoCasado-Coterillo, ClaraNanami, NorimichiCasalini, R.Nand, AshveenCaschera, DanielaNandwana, VikasCaseri, WalterNarayanan, GaneshCasettari, LucaNaumann, StefanCastell, PereNaumov, AntonCastiglione, KathrinNavacerrada, María De Los ÁngelesCastiglione, FrancaNavarro, Francisco JavierCastñeiras, AlfonsoNazare, ShonaliCastro, NathanNegahdar, LeilaCausa, FilippoNejati, SiamakCavallaro, GiuseppeNelatury, SudarshanCavicchi, KevinNeri, SimonaCefalas, A.C.Nesterenko, Vitali F.Celasco, EdvigeNeufurth, MeikCelik, EmrahNeugebauer, DorotaCelzard, AlainNeves, MariaCendrowski, KrzysztofNgo, TrucCennamo, NunzioNguyen, Ngoc A.Cerbu, CameliaNicasio, Maria CarmenCesano, FedericoNicolay, RenaudCha, Sung WoonNicoletta, Fiore PasqualeCha, ChaenyungNielsen, Line HagnerChae, MichaelNieminen, KaarloChalioris, ConstantinNieto, DanielChamonine, MikhailNieuwendaal, Ryan C.Chan, Yi-TsuNikkhah-Moshaie, RoozbehChan, Hon FaiNikolakakis, IoannisChandroth Pannian, JishaNirichan, Sanoj RejinoldChang, Chih-YuNischang, IvoChang, JiyoungNishi, KengoChao, HuikuanNishida, MasakazuChaouch, MounirNishikiori, HiromasaCharas, AnaNishikitani, YoshinoriChaubet, FrédéricNishimoto, YoshioChavez, FermanNishiyabu, RyuheiChavez-Angel, EmigdioNisticò, RobertoChecchetto, RiccardoNiu, HaitaoChen, MingshengNobile, LucioChen, Yong-SongNoor, NuruzzamanChen, Chuh-YungNorrman, JensChen, LingxinNouranian, SasanChen, ZhihangNovak, IgorChen, JeffNuhn, LutzChen, JiahaoObata, KotaroChen, JihuaOda, YukariChen, Po-chiaOdelius, KarinChen, I WenOesterschulze, EgbertChen, XiaogangOgam, ErickChen, TianyiOgawa, NorikoChen, JinhuaOgino, ToshioChen, Lung-ChienOgnyanov, ManolChen, JunOh, Dongyeop X.Chen, Jem-KunOh, Kyung WhaChen, ChiachungOhara, KeishiChen, Ruei-TangOhta, YoshihiroChen, Hui-YuOjstršek, AlenkaChen, Yi-ChunOkamoto, MasamiChen, Zhi-GangOkuzaki, HidenoriChen, XiangzhongOliaei, ErfanChen, YijiaOliva, LeoneChen, Kuo-YuOliveira, SaraChen, ChunhuiOller, EvaChen, Jhy-DerOmiya, MasakiChen, TaoOrbulov, ImreChen, LinOrlandini, EnzoCheng, H. N.Orsi, MarioCheng, PeiOrte, AngelCheng, H.N.Ortenzi, Marco A.Cheng, Chih-ChiaOrtiz, AlfredoCheng, ZhiqiangOsakada, KohtaroCheong, In WooOsswald, TimChereddy, Kiran KumarÖsterholm, AnnaChiadò, AlessandroOtero, MartaChiang, Long Y.Otero-Espinar, Francisco JavierChiang, Chin LungOtsuki, JoeChiang, Yi-TingOttonelli, MassimoChiavaioli, FrancescoOuyang, JianyongChickov, BorisOuyang, LiangqiChiessi, EsterOwen, ShawnChiolerio, AlessandroOzaki, RyotaroChiu, Kuan-ChengPaavilainen, SamiChmielarz, PawełPaci, MaurizioCho, SunghunPaczesny, JanCho, Dong-WooPadil, Vinod V. T.Cho, In SunPadilla, JavierChockchaisawasdee, SuwimolPal, SudiptoChoi, Jae-HongPaladini, FedericaChoi, Seung-BokPalamà, IlariaChoinopoulos, IoannisPalermo, EdmundChong, ParksonPalla, FrancoChorazy, SzymonPalumbo, FabioChou, Shih-FengPan, HaihuaChremos, AlexandrosPan, GuoqingChrissopoulou, KiriakiPan, LijiaChristova, DarinkaPan, QinChruściel, Jerzy J.Panagiotopoulos, Ioannis A.Chu, FuxiangPandey, TribhuwanChuang, Er-YuanPandini, StefanoChüeh, Anderly C.Panicker, RajeshChun, Heung JaePanico, AntonioChung, Sheng-HengPantani, RobertoChung, Chan-MoonPantano, MariaChung, Hyun-JoongPantelides, Chris P.Ciambella, JacopoPanzarasa, GuidoCiani, BarbaraPaolino, MarcoCiardelli, FrancescoPapaefstathiou, Giannis S.Cicala, GianlucaPapageorgiou, George Z.Cicchi, StefanoPapagiannopoulos, AristeidisCidade, Maria TeresaParaskevopoulou, PatrinaCieplak, MarekParedes, José ManuelCiesielski, MichaelParisi, SalvatoreCiriza, JesusParisi, FilippoCochis, AndreaParisini, EmilioCoelhoso, IsabelPark, Soo-JinColeman, Anthony WilliamPark, Ji-WonCollins, Maurice N.Park, Jong-SeungColombi, PierluigiPark, JuhyunComerford, JamesPark, Jong-SeokConsoli, AntonioPark, HansooContreras-Cáceres, RafaelPark, Byung-DaeCordeiro, Nereida M.A.Park, Young IlCorigliano, AlbertoPark, Chan HoCornell, B.Park, JeyoungCornil, JérômePartsch, HugoCorradi, MarcoParvinzadeh Gashti, MazeyarCorreia, Ilídio J.Paso, KristoferCortese, BarbaraPasparakis, GeorgiosCosma, PinalysaPasqua, LuigiCosta, Carlos M.Passaglia, ElisaCosta de Morais, Rui ManuelPassos, HelenaCouce, María D.Pastero, LindaCoutinho, Paulo José GomesPastore, RobertoCovas, JosePaszkiewicz, SandraCowan, Matthew GreigPatel, ManuCragg, PeterPater, ZbigniewCran, Marlene J.Patil, Sunil A.Craw, PascalPatra, NiranjanCristea, CeciliaPatrignani, FrancescaCroce, Anna CletaPatterson, JenniferCroll, Andrew B.Patterson, BrianCrucho, CarinaPaul, Suvash ChandraCruy Silva, RodolfoPavlopoulou, EleniCsoka, LeventePawar, RadheshyamCui, HaitaoPawlikowski, MarekCui, WenguoPawlowski, MichalCui, FangdaPazur, R. J.Culver, HeidiPeer, AkshitCybulska, JustynaPegoretti, AlessandroCyganowski, PiotrPellis, AlessandroCzarnecka-Komorowska, DorotaPenczek, StanislawD’Anna, FrancescaPenkov, Oleksiy V.Da Silva, MarceloPerazzo, AntonioD’accolti, LuciaPeresín, Maria SoledadDagdeviren, CananPérez-Juste, JorgeDahle, SebastianPergushov, Dmitry V.Dai, LizongPerinelli, Diego RomanoDala, LaurentPerlepes, Spyros PDalla Rosa, MarcoPerrin, LaraDa’na, EnshirahPerteghella, SaraDanko, MartinPestov, AlexandrDargaville, TimPetcu, CristianD’Arienzo, MassimilianoPeter, FranciscDarinskii, Anatoly A.Peter, KatherineDarling, SethPeterson, BrandonDas, SanjibPetkova, DianaDas, OisikPham, JonathanDash, BirajaPham, TungDaugevičius, MykolasPhan, Hoang-PhuongDavis, FrankPhoenix, S. LeighDavis, FredPhuoc, Nghia TruongDawes, JudithPiedade, Ana PaulaDawn, ArnabPielichowski, KrzysztofDe Aza, Piedad N.Pierini, FilippoDe Bonis, AngelaPieta, PiotrDe Caro, VivianaPietrasik, JoannaDe Falco, GianluigiPifferi, ValentinaDe Gonzalo, GonzaloPiluso, SusannaDe Grooth, JorisPilyugina, EkaterinaDe La Flor, SilviaPinho, EvaDe Laporte, LauraPinho, PedroDe Masi, LuigiPiñol, MilagrosDe Simone, Marco ClaudioPinteala, MarianaDebby, GawlittaPinto, DeesyDebéda, HélènePinto, Artur M.Deghani, FaribaPiorkowska-Galeska, EwaDekel, Dario R.Pirnat, KlemenDelaney, ColmPirzada, TahiraDelattre, CédricPispas, StergiosDelbe, KarlPissis, PolycarposDelmotte, LucPistone, AlessandroDelorme, NicolasPitsikalis, MarinosDelpouve, NicolasPitto-Barry, AnaïsDembsey, Nicholas APiunova, VictoriaDeng, ZiweiPizzi, AntonioDeng, HuaPizzolanti, GiuseppeDergunov, Sergey A.Plamper, FelixD’Errico, GerardinoPojman, John A.Deuss, PeterPokorny, MarekDevaraj, AishwaryaPoletto, MatheusDewapriya, M.A.N.Polimeni, AntonellaDhakal, DipeshPomerantz, Andrew E.Dhakal, RajendraPons, JosefinaDhanasekar, ManickaPoole-Warren, LauraDhindsa, Kulwinder SinghPopielarz, RomanD’hooge, Dagmar R.Postnikov, PavelDi Girolamo, RoccoPotapov, AndreiDi Maio, LucianoPourrahimi, Amir MasoudDi Noia, Luigi PioPradhan, ShantanuDi Sarli, ValeriaPradhan, NirakarDias, JulianaPraprotnik, MatejDias, Rolando CSPresa, AlejandroDiban, NazelyPretsch, ThorstenDickey, MichaelProcek, MarcinDiddens, DiddoProchazka, KarelDiep, Hung T.Profire, LenutaDíez-Pascual, Ana MaríaPron, HervéDimos, KonstantinosProrokova, N. P.Ding, JianxunProto, Andrea RosarioDing, Wei DanPruna, Alina IulianaDing, ChunbangPrzybylek, PiotrDing, JunjunPu, HuiDing, LiyunPucci, AndreaDixon, DavidPuchalski, MichalDjenizian, ThierryPuglia, DeboraDjeziri, Mohand ArabPuiggalí, JordiDobrzynski, PiotrPullano, Salvatore AndreaDodiuk, HannaPunzo, FrancescoDöhler, DianaPuppi, DarioDolez, PatriciaPuranik, AmeyDomb, AviPuttreddy, RakeshDomenici, ValentinaPyo, SukhoonDominguez, CesarQi, GuangyanDong, WeiQian, YongDong, ChensongQiang, ZheDong, YingchaoQin, GuanDorado, ChristinaQin, JunshengDorfman, KevinQing, LimingDoroudiani, SaeedQuynh Hoa, LeDou, JinboRabczuk, TimonDräger, GeraldRae, P.J.Drevenšek-Olenik, IrenaRaeis Hosseini, NiloufarDrioli, EnricoRaffa, PatrizioDrummond, CarlosRafiee, MohammadD’Sa, RaechelleRaftopoulos, KonstantinosDu, XushengRaga, Sonia R.Du, HongjianRagaert, KimDu, FanfanRagauskas, Arthur J.Du, JuanjuanRagona, LauraDuan, BinRagusa, AndreaDubey, AshishRahimabady, MojtabaDufossé, LaurentRahman, Mohammad MizanurDumée, LudovicRahme, KamilDumitran, Laurenţiu MariusRais, RanaDunky, ManfredRamanavicius, ArunasDuong, HaiminhRamiasa, MelanieDurazzo, AlessandraRamos, JavierDurmus, AliRamsay, BruceDurner, WolfgangRana, DipakDutta, BiswanathRao, SmithaDutta, AbhishekRao, AshitDuty, ChadRao, AgnėDziubla, ThomasRao, AshwinDzolic, ZoranRaposo, MariaEbara, MitsuhiroRapson, Trevor D.Eddy, LiyantoRaspanti, MarioEdirisinghe, MohanRatke, LorenzEduok, UbongRaucci, Maria GraziaEhm, ChristianRault, FrançoisEhrlich, HermannRavelli, DavideEhrmann, AndreaRebecca, BraslauEhtemam-Haghighi, ShimaRebollar, EstherEjfler, JolantaRecek, NinaEkambaram, ManikandanReece, MikeEkielski, AdamReglero Ruiz, José AntonioEklund, PerReilly, GwendolenElder, RobertReimhult, ErikEl-hadi, Ahmed M.Reiter, GünterElhag, SamiRen, KaixuanElias, Anastasia L.Ren, JizhongElSherbiny, IbrahimRennhofer, HaraldEmilsson, GustavReyes-Ortega, FelisaErbas, AykutRezaei-Gomari, SinaErdem, EmreRhee, Jin-KyuEric, TillmanRiba, Jordi-RogerErythropel, HannoRibeiro, Maria C.S.Escobedo-Lucea, CarmenRibeiro, ClarisseEscorihuela, JorgeRicci, GiovanniEscudero, NuriaRiccò, RaffaeleEslami, HosseinRice, Cynthia AnnEspinach, Francesc XavierRichardson, JosephEsposito, Emilio XavierRichtera, LukášEstelle, PatriceRider, AndrewEsteves, Bruno MRiegler, MartinEstrany, FrancescRiess, GisbertEtxabide, AlaitzRiggleman, RobertEtxebarria, JesúsRimoldi, LucaEugenio, María E.Ristori, SandraEvtugyn, GennadyRiziotis, ChristosFaccio, GretaRoberto, DominiqueFagerstedt, KurtRobinson, SaraFan, ChenguangRocchigiani, LucaFan, WeizhengRodrigues, Alirio EgidioFan, WeiRodriguez, AlejandroFang, HuagaoRodriguez Lorenzo, Luis MariaFare, SilviaRodríguez-López, JuliánFaria Albanese, Jimmy AlexanderRodriguez-Ropero, FranciscoFarkaš, PavolRogers, Simon A.Farris, StefanoRomano, ValerioFarrugia, BrookeRomero, PedroFasano, VitoRonca, SaraFeczkó, TivadarRondeau-Gagné, SimonFederici, StefaniaRonen, AvnerFei, BinRoppolo, IgnazioFeld, ArturRosa, Angela Daniela LaFellows, ChrisRosato, AntonioFeng, ChuangRoseti, LiviaFeng, HongboRosicka-Kaczmarek, JustynaFeng, Chuan-LiangRotaru, AndreiFerhan, Abdul RahimRoth, Stephan V.Fernandes, Emanuel M.Roth, BernhardFernandes, IsabelRoth, Peter J.Fernandes, Susete NogueiraRousakis, TheodorosFernandes, CarlaRoy, SwarupFernandes, Mariana Sofia PeixotoRoy, SagarFernandes, EmanuelRoyal, GuyFernández, RobertoRozga, PawelFernández-d’Arlas, BorjaRubio, Ramón G.Fernández-Francos, XavierRudawska, AnnaFernández-García, MartaRudick, JonathanFernández-Lafuente, RobertoRudykh, StephanFerrandiz Bou, SantiagoRuffino, FrancescoFerruti, PaoloRuiz, Jordi-Roger RibaFigueira, Rita BacelarRuiz, M. PilarFilgueira, CarlyRuiz Rubio, LeireFilip, PetrRuiz-García, AlejandroFinkenstadt, VictoriaRuiz-Rosas, Ramiro RafaelFiori, StefanoRumyantseva, Marina N.Firlak, MelikeRupprich, MarcoFischer, DagmarRüscher, ClausFlandin, LionelRusso, SaverioFlege, StefanRyan, James G.Fliedel, ChristopheRydz, JoannaFlorczyk, Stephen J.Sá, JacintoFodor, CsabaSabater I Serra, RoserFologea, DanielSabel, TinaFonseca, Ana ClotildeSaçak, MehmetForaboschi, PaoloSacco, RiccardoForeman, JoelSada, KazukiForget, AurelienSahakaro, K.Formela, KrzysztofSahariah, PriyankaFortea-Verdejo, MartaSai, HiroakiFossum, EricSaid Schicchi, DiegoFoster, Christopher FosterSaielli, GiacomoFrancesco, NegroSainsbury, TobyFranco, José M.Saito, TakuyaFrancolini, IolandaSaiz, FernanFranko, AndrasSakaue, TakahiroFrazier, CharlesSalaoru, IuliaFrediani, PieroSalas, CarlosFreire, FélixSalas-de La Cruz, DavidFricke, KatjaSalaun, FabienFrielinghaus, HenrichSalim, NisaFrigione, MariaenricaSalmeia, Khalifah A.Frihart, CharlesSambuy, YulaFroeyen, MatheusSameoto, DanFromm, KatharinaSamia, BenmansourFu, XiaotingSamyn, FabienneFu, TengfeiSanchez, Ana BelenFujita, Shin-ichiroSánchez, ManuelFukuda, TakeshiSánchez-García, DavidFuoco, TizianaSanchís, María JesúsFuoco, AlessioSanchiz, JoaquinFurlani, MaurizioSandri, GiuseppinaFurusawa, HiroyukiSandwall, PeterFusco, CaterinaSanjust, EnricoFustin, Charles-AndréSankarasubramanian, ShrihariGaan, SabyasachiSantamaria-Echart, ArantzazuGahleitner, MarkusSantana, Orlando O.Gaitzsch, JensSantoni, Marie-PierreGajjar, ChiragSantoni, IlariaGaku, FukuharaSantos, LúciaGala, Rikhav PSantos, DiogoGaldi, Giovanni P.Sanz, MikelGalkin, MaximSanz Garcia, AndresGallei, MarkusSapi, AndrasGamez-Perez, JoseSapino, SimonaGan, Yong X.Sardar, SamimGanewatta, MitraSartorio, CamilloGanster, JohannesSasai, YasushiGao, HuileSashiwa, HitoshiGao, PeiyuanSato, KimiyasuGao, YangSato, KatsuhikoGao, YongfengSato, YuichiroGao, HaiyangSatriano, CristinaGao, ZheSavagatrup, SucholGao, YunxiangSavi, PatriziaGarbovskiy, YuriyScala, AngelaGarcía, NuriaScalici, TommasoGarcia-Deibe, AnaŠčetar, MarioGarcía-González, Carlos A.Schacher, FelixGarcía-Martinez, Joaquín C.Schaefer, JenniferGarcía-Martínez, Jesús-MaríaScharnagl, NicoGardan, JulienSchartel, BernhardGavara, RafaelScheler, UlrichGazzano, MassimoSchirhagl, RomanaGe, TingSchirp, ClaudiaGeiger, Franz M.Schlater, GuyGeise, GeoffreySchlosser, DietmarGellerstedt, GöranSchmid, ManfredGentile, GennaroSchmid, MarkusGentile, PiergiorgioSchmid, JochenGeorge, Thomas F.Schmidt, BernhardGeorgiadou, StellaSchmitt, MichaelGeorgopanos, ProkopiosSchweizer, ThomasGervais, FrançoisScoponi, MarcoGhahremaninezhad, AliScotognella, FrancescoGhalei, BehnamScrano, LauraGhandi, KhashayarSedláček, J.Gharam, AhmedSedlačík, MichalGhobadi, EhsanSeidel, Rüdiger W.Ghoneim, MohamedSeki, TakahiroGhosh, DebanjanaSelyanchyn, RomanGhoshal, SushantaSemenyuk, PavelGiansanti, LuisaSenatov, Fedor S.Gierszewska, MagdalenaSeo, Dae-ShikGigli, MatteoSeo, Young-SooGimenez-Pinto, VianneySequeira, SílviaGindl-Altmutter, WolfgangSerier-Brault, HélèneGiner-Casares, Juan J.Serpooshan, VahidGiraud, StephaneSerra, AngelsGizdavic-Nikolaidis, MarijaSerrano-Aroca, ÁngelGlomm, Wilhelm RSeydel, TiloGloria, AntonioSeyedin, ShayanGlover, Carol F.Shafiei, MahnazGlüsen, AndreasShafiei, AliGockenbach, ErnstShamonin, MikhailGoding, JosefShanks, Robert AGoh, Suat HongSharma, Piyush SindhuGolaszewski, JacekSharma, AjitGomes, Ana P.Shau, Min DaGomez, ClaraShavandi, AminGómez-Ruiz, SantiagoShehata, NaderGómez-Soberón, José ManuelShekhtman, AlexanderGon, MasayukiShen, Yu-FangGonçalves, Lidia M DSher, PraveenGong, HuaShestakov, MikhailGonzalez, JoaquinShi, XiaoyangGonzalez, ZoraidaShi, YushengGonzález, IsraelShi, KaiyuanGonzález, SergioShi, WenboGonzález-Fonteboa, BelénShi, HengchongGonzález-Pérez, José A.Shibasaki, YujiGopalan, Sai AnandShimada, ShigeruGordiichuk, PavloShin, Jae SupGoreham, ReneeShinde, SudhirkumarGorrasi, GiulianaShinkai, KoichiGoseki, RaitaShiono, TakeshiGoswami, MonojoyShipp, Devon A.Gozdzicka-Jozefiak, AnnaShirvanimoghaddam, KamyarGracia, IgnacioShityakov, SergeyGraiff, ClaudiaShoji, YoshiakiGranet, RobertShou, DahuaGrau, GerdShou, WanGravalidis, ChristoforosShrestha, Lok KumarGreco, Antonio Siller, Hector R.Green, Nicola H.Simocko, ChesterGrest, Gary S.Simoncic, BarbaraGriffini, GianmarcoSimonescu, Claudia MariaGrijalvo, SantiagoSims, Ian M.Gritsan, Nina P.Singh, Jitendra KumarGrivel, Jean-ClaudeSinha, JasmineGroch, PawełSinha, AbhijeetGu, YuweiSinha Ray, SumanGu, XiaodanSiracusa, ValentinaGuan, WenjianSirvio, JuhoGuazzelli, LorenzoSixta, HerbertGüemes, AlfredoSkorik, Yury A.Guerra, GaetanoSloop, JosephGuerriero, GeaSmiatek, JensGuessasma, SofianeSmith Callahan, Laura A.Guex, Anne GéraldineSnyder, NicoleGuigo, NathanaelSoares, PaulaGulgunje, PrabhakarSobiesiak, MagdalenaGuo, ZhanhuSobolewska, AnnaGuo, QiuquanSoccio, MichelinaGuo, ZhanyongSödergård, AndersGuo, Zhao-XiaSohrabpoor, HamedGuo, HuiSoin, NavneetGupta, AnjuSola, LauraGupta, PrachiSon, Seung UkGupta, MohitSon, DongheeGupta, KajalSong, ZiyuanGupta, SanjuSong, KenanGurikov, PavelSong, HengxuGutekunst, Will R.Song, JuhaGutierrez, JunkalSonnier, RodolpheGutiérrez-Climente, RaquelSontakke, Atul DGutmann, Jochen S.Sorrentino, AndreaGuzmán Solís, EduardoSorrentino, LuigiHabas, Jean-PierreSorrentino, LucaHabisreuther, TobiasSotta, PaulHadavinia, HomayounSoulat, DamienHaddadi, KamelSousa, AnaHadipour, AfshinSpanova, AlenaHadjiargyrou, MichaelSparavigna, Amelia CarolinaHadwiger, LeeSperanza, VitoHafuka, AkiraSperanzini, EmanuelaHager, Martin D.Spina, RobertoHalada, GarySrebnik, SimchaHamad, KotibaSrivastava, SaurabhHammerl, Jens AndreSrubar, Wil V.Hampp, NorbertStachewich, UrszulaHamzehlou, ShaghayeghStaicu, TeodoraHan, Jong WonStanciu, Mariana DomnicaHan, YousooStarowicz, MałgorzataHan, JingStasiak, AndrzejHandge, Ulrich A.Stassen, IvoHanif, AsadSteinfeldt, NorbertHanyková, LenkaStekovic, DejanHao, AiyouStelescu, Maria DanielaHaouas, MohamedStephanou, Pavlos S.Haponiuk, JózefSteup, MartinHaque, Bazle Z.Stevanovic, TatjanaHardy, John G.Stevens, TimHarman, DavidStewart, BeverlyHarničárová, MartaStöckelhuber, Klaus WernerHarnois, MaximeStokke, Bjørn TorgerHasani, MerimaStolarczyk, AgnieszkaHasegawa, MasatoshiStoleru, ElenaHashidzume, AkihitoStone, KariHashizume, MineoStout, David A.Haspel, NuritStoyan, DietrichHatzignatiou, Dimitrios GeorgiosStoychev, GeorgiHaurie Ibarra, LaiaStrankowski, MichałHaut Donahue, TammyStratakis, EmmanuelHawinkels, LukasStride, John ArronHawkett, BrianStrzemiecka, BeataHayakawa, TohruStull, Jamie A.Hayashi, ShotaroStumpe, JoachimHe, WenhanStylianakis, MinasHe, GuangweiSu, ZhiqiangHe, WeidongSu, An-ChungHe, HongliangSu, Chia-HaoHe, ZhongqiSu Jeong, HyeonHee Na, JunSubramaniam, PrasadHeeley, Ellen LSuchanicz, JanHegmann, EldaSudo, AtsushiHeim, FredericSue, Hung-JueHeine, ElisabethSugimoto, TomokoHejna, AleksanderSuh, Won HyukHellweg, ThomasSujecki, SlawomirHelm, Christiane A.Sukhishvili, SvetlanaHemraz, Usha D.Summerscales, JohnHenderson, Luke C.Sun, HongliHenniges, UteSun, HaoHenriksen, Niel M.Sun, Bao AnHermanová, SoňaSun, LuyiHernandez, RebecaSun, Ya-SenHeydari Gharahcheshmeh, MeysamSun, RuncangHideto, MinamiSun, YongleHiejima, YusukeSun, JingjingHildreth, BlakeSun, YingjieHill, Josephine MarySun, F.W.Hippler, RainerSun, DiHirva, PipsaSun, QingHizal, GürkanSunatsuki, YukinariHo, Mei-LinSundaram, PaulHo, Wen-JengSundarrajan, SubramanianHolmes, Natalie P.Sundqvist, BertilHonda, YoshitomoSuraneni, PrannoyHong, LiangSusmel, SabinaHong, Suck WonSvoboda, PetrHong, SukjoonSweetman, MartinHorrocks, A. RichardSwindle-Reilly, Katelyn E.Horvai, GeorgeSylvestre, AlainHorváth, JuditSyu, Mei-jywanHoskins, ClareSzczurek, AndrzejHosseini Nejad, ElhamSzekely, GyorgyHosseinpourpia, RezaSzewczyk, RomanHou, JingweiSznitowska, MałgorzataHoujou, HirohikoSzolnoki, BeátaHowell, BobSzostak, MarekHrnčič, Maša KnezSzumny, Antoni JacekHsiao, Pai-YiSzymańska, EmiliaHsiao, Sheng-HueiSzymanska-Chargot, M.Hsieh, Chien TengSzymanski, RyszardHsu, Fu-YinTabakovic, AmirHsu, Steve Lien-ChungTabatabaei, AlirezaHsu, Hsiu-FuTábi, TamásHu, XiaoTachibana, YuyaHu, Shang-HsiuTada, NaoyaHu, YangTaddei, PaolaHu, Ching-YaoTahergorabi, RezaHu, JinlianTai-Shung Chung, NealHu, JinmingTajiri, KazuyaHu, GuangTakano, AtsushiHua, TaoTakano, ToshiyukiHuang, JingTakemoto, HiroyasuHuang, WeiminTakeno, HiroyukiHuang, YuchengTakeuchi, DaisukeHuang, ChangshuiTalapaneni, Siddulu NaiduHuang, PeihuaTamás, TörökHuang, QijinTanaka, TomonariHuang, Chih-FengTanaka, KazuoHuang, WenTanaka, RyoHuang, YugangTananaiko, OksanaHuang, Chun-JenTang, FeiHuang, Chi-HsienTang, YouhongHuang, Chun-YungTang, JieHuang, FeiheTaniike, ToshiakiHuerta-Angeles, GloriaTao, FeifeiHuining, HuiningTarrés, QuimHutchinson, Robin A.Tatar, JovanHuynh, VienTaubert, AndreasHwang, Ye-JinTaylor, SpencerHwang, YongsungTelegdi, JuditHwang, Seok-HoTemple, HenryIandolo, DonataTena, AlbertoIatrou, HermisTenorio-Alfonso, AdriánIbrahim, AhmedTeramoto, YoshikuniIconaru, SimonaTeramoto, NaozumiIdris, Maizlinda IzwanaTercjaka, AgnieszkaIglesias, EmiliaTervoort, TheoIkegami, MasashiThakur, Vijay KumarIlg, PatrickThang, Tran QuyetIllangakoon, UpulithaTheres Baby, TessyIna, MariaThibault, JulesInomata, KatsuhiroThomas, T.J.Iordanskii, AlexeyThomas, SamuelIrie, MasaoThrimawithana, ThiliniIshige, RyoheiThrivikraman, GreeshmaIshikawa, RyoThünemann, AndreasIshikawa, RyutaTibbitt, MarkIshizone, TakashiTiera, MarcioIslamoglu, TimurTilley, MichaelIsmail, AhmedTimofeev, Ivan V.Iso, YoshikiTiraferri, AlbertoIta, KevinToca-Herrera, JoseIto, ShingoTognetti, AlessandroIto, SeigoTolstoy, Peter M.Ivask, AngelaTomaszewska, EwaJacak, JarosławTomppo, LauraJaczewski, MariuszTondi, GianlucaJaffrezic-Renault, NicoleTonelli, Alan EdwardJaiswal, SwarnaTorino, EnzaJajam, Kailash C.Torre, Maria LuisaJamróz, PiotrTorres, CristianaJanas, DawidTosello, GuidoJanczarek, MonicaToshio, KoizumiJang, Kyung-InTretsiakova-McNally, SvetlanaJanovák, LászlóTrník, AntonJasion, GregoryTrotta, FrancescoJastrzab, RenataTrovato, FabioJeeyoung, YooTruong, VinhJefferis, StephanTrusso Sfrazzetto, GiuseppeJelliss, Paul A.Tsai, Chia-HungJensen, Ole M.Tsakalof, AndreasJeon, Jong-RokTsarkova, Larisa A.Jeon, Ju-WonTseng, Hui-hsinJeong, SunghoonTsitsilianis, ConstantinosJeong, Hoon EuiTsoi, JamesJeong, HyangsooTsuchiya, KousukeJermakowicz-Bartkowiak, DorotaTsugawa, WakakoJesionowski, TeofilTsuji, HidetoJeziorska, ReginaTsujimoto, TakashiJia, ZhongfanTsukada, SatoruJia, DiTsutsui, MakusuJian, RongkunTsuyumoto, IsaoJiang, BingyanTunesi, MartaJiang, JiangTwigg, PeterJiang, TaoTyliszczak, BożenaJiang, LaiTylkowski, BartoszJiang, ShaohuaTzounis, LazarosJiang, LiuweiUchida, YoshiakiJiao, XuechenUdalov, Oleg GeorgievichJicsinszky, LászlóUddin, MohammadJin, KailongUemura, KazuhiroJin, CongruiUeta, IkuoJin, JiefuUlanski, PiotrJin, Hyeong MinUllah, AmanJirsák, OldřichUlven, Chad A.João, BenevidesUmer, RehanJofre-Reche, Jose A.Umeton, CesareJohannes, BrendelUneyama, TakashiJohnson, Blake N.Unno, MasafumiJoly-Duhamel, ChristineUnterreiner, Andreas-NeilJones, ChrisUrbanc, BrigitaJong, Wen-RenUsui, KenjiJordan, AlexUyama, HiroshiJoshi, VijayaVagin, MikhailJoshi, KaushikVago, RiccardoJoshi, NiravkumarVahabi, HenriJu, HeongkyuVaidyanathan, SriramJuárez, Jaime JavierValentini, LucaJung, Ho-SupVamanu, EmanuelJung, JaehanVan Herwijnen, Hendrikus W. G.Jung, HyunVan Rijn, PatrickJuodkazis, SauliusVan Steenberge, Paul Hm M.Jurić, MarijanaVankayala, RavirajJuszczak, LesławVarfolomeev, Mikhail A.Kadimisetty, KarteekVargas, ZulemaKadokawa, Jun-ichiVarghese Chacko, JenuKadumudi, Firoz BabuVasile, CorneliaKafarski, PawelVaughan, AlunKahlert, HeikeVautard, FredericKahr, BartVázquez-López, Ezequiel M.Kai Chua, CheeVecchione, RaffaeleKakarla, Raghava ReddyVega Gutierrez, SarathKalantar-Zadeh, KouroshVenkataraman, ShrinivasKali, GergelyVenkatesan, JayachandranKallitsis, Joannis K.Venkateshvaran, DeepakKamamichi, NorihiroVerdugo-Escamilla, CristobalKamdem, Donatien PascalVerma, SannyKaminski, KamilVernardou, DimitraKan, Chi-waiVerstappen, PieterKanakubo, ToshiyukiVert, MichelKanazawa, ArihiroVidal-Verdú, FernandoKandelbauer, AndreasVilloria Sáez, PaolaKanduc, MatejViñas, MiguelKang, XinVirnau, PeterKang, HyoVirta, PasiKang, Hyun WookVisai, LiviaKanik, MehmetViswanadam, GouthamKao, Chai-LinVitale, AlessandraKappera, RajeshVlachopoulos, JohnKarahan Toprakci, Hatice AylinVlachou, MarilenaKaralekas, DimitrisVohra, VarunKarapanagiotis, IoannisVoicu, SIKarger-Kocsis, JózsefVoigtmann, ThomasKari, LeifVoliani, ValerioKarlsson, ChristofferVolker, KörstgensKaruppasamy, KannanVollebregt, StenKaseem, MosabVorotyntsev, IlyaKasem, Kasem K.Voué, MichelKashi, SimaVouyiouka, StamatinaKassama, LaminVrana, Nihal EnginKatashima, TakuyaVrsaljko, DomagojKatepalli, HariVu Bac, NamKatunin, AndrzejVuluga, ZinaKaur, Aman PreetVyazovkin, SergeyKaushik, Nagendra K.Wach, Radosław A.Kawakita, HidetakaWagland, StuartKawamura, IzuruWagner, KlaudiaKawamura, AkifumiWalach, WojciechKazuhiro, ShikinakaWaldmann, DanièleKehl, FlorianWałęsa-Chorab, MonikaKéki, SándorWang, QingQingKeplinger, TobiasWang, ZhongchangKerch, GarryWang, Jian-WenKeul, HelmutWang, XiaofengKevadiya, BhaveshWang, Yi-ChengKevin, Ge QiWang, PangpangKhairulina, KaterynaWang, HaoyuKhan, Omar F.Wang, QinyiKhan, AnzarWang, BaominKhanbareh, HamidehWang, YuKhandaker, MorshedWang, MingchaoKhang, GilsonWang, XiaoguangKhansur, Neamul HayetWang, LeiKhapli, SachinWang, DongKharkar, PrathameshWang, Chih-ChiehKhayet, MohamedWang, YunxiaoKhokhlov, AlexeiWang, Tzu-WeiKhotimchenko, Maxim Y.Wang, Xiao-YongKhulbe, K. C.Wang, LuKhurshid, ZohaibWang, ChenKiefer, RudolfWang, ZuoweiKihara, NobuhiroWang, ZhaoKihara, Shin-ichiWang, Jian-GanKim, Gue-hyunWang, XinKim, YonghoWang, BoKim, JongseongWang, Yun-CheKim, Won KyuWang, LingKim, WonhoWang, De-YiKim, Bo-HyunWang, QiuguKim, Hak-YongWang, WenyiKim, Nam KyeunWang, Michael CaiKim, B.K.Wang, KaiKim, Sang JaeWang, HuimingKim, Young-KwanWang, YanKim, JaheonWang, Yen-ZenKim, Woo SooWang, JingtaoKim, KyoungokWang, Jun KitKim, Hyug-HanWarszyński, PiotrKim, JooyounWeber, DanielKim, KukjooWedel, ArminKim, Young DokWEI, JIAKim, Hyun-JoongWei, KongchangKim, Il TaeWei, GangKim, DonginWei, ZhongbaoKim, JoohoonWei, KayaKimura, KunioWei, Yu-HongKimura, ShunsakuWei, QiangKing, LaurieWei, QingshuoKinoshita, TakumiWei, RenboKinthada, RamakumarWei, BingKiparissides, CostasWei, JunchaoKirillova, AlinaWen, JialongKishimura, AkihiroWeng, ZhihuanKiss, AnnaWhelligan, DanielKitazawa, TakafumiWhitford, Paul CKlajnert-Maculewicz, BarbaraWhitmer, JonathanKlonos, PanagiotisWibowo, DavidKnittel, DominiqueWieckiewicz, MieszkoKnupp, GerdWieczorek, Władysław G.Knutsen, SveinWieleba, WojciechKobayashi, YoshiroWießner, SvenKoduru, Janardhan ReddyWilczyński, KrzysztofKoeckelberghs, GuyWillems, WimKoh, Leng-DueiWilmer, ChristopherKohri, MichinariWilson, LeeKohsaka, YasuhiroWinnacker, MalteKoibuchi, HiroshiWińska, KatarzynaKoike, KenzoWinter, AndreasKoizumi, HiroyasuWischke, ChristianKokado, KentaWitt, KatarzynaKoller, MartinWohl, Christopher J.Konda Gokuldoss, PrashanthWohlert, JakobKondo, MioWolcott, Michael PKong, Ling BingWolny, StefanKong, XiangruiWong, Michael YinKong, LingxueWoo, EamorKonieczny, JaroslawWoo Jin, JungKonkolewicz, DominikWoodward, Robert T.Kontoni, Denise-PenelopeWoodward, CliffordKontou, EviWu, JianshengKontturi, EeroWu, YonggangKootala, SujitWu, XiangfaKopel, PavelWu, YunlongKopjar, MirelaWu, Tzi YiKortaberria, GalderWu, Jing-YunKortschot, Mark T.Wu, NingjingKorzhikov-Vlakh, ViktorWu, JianboKošak, AljošaWu, Hung-ChinKoseva, NeliWu, HongwuKossack, WilhelmWu, Jyh-HorngKostopoulos, VassilisWu, Ming-ChungKosuke, SugawaWu, ZongquanKotsuchibashi, YoheiWu, Yu-TseKoumoulos, Elias P.Wycisk, RyszardKowalczuk, Marek M.Wyman, IanKozakiewicz, AnnaWysokowski, MarcinKozioł, MateuszXavier, José Manuel CardosoKozliak, Evguenii I.Xi, WeixianKozłowska, MariolaXi, LiKremer, FriedrichXia, WenjieKressler, JoergXia, ChangleiKroger, MartinXiang, YuanKrol, JanXiao, RuiKról-Kilińska, Żanetaxiao, boqiKruczala, KrzysztofXie, HongfengKruth, Jean-PierreXie, KunpengKu, Bon-CheolXin, HaiKuang, HuihuiXing, Jin-FengKubo, MasatakaXu, YongKubo, IzumiXu, TaoKucinska-Lipka, JustynaXu, BinKuckling, DirkXu, YitingKucuk, IsrafilXu, ChunfuKugler, SzymonXu, BingangKujawa, JoannaXu, YingjieKujawska, MałgorzataXue, JiajiaKujawski, WojciechXue, XiaoqiangKukla, ChristianXue, JinkaiKulshreshtha, PrashantXue, ZhigangKulzer, FlorianYadav, SushmaKumar, PrasunYadavalli, Nataraja SekharKumar, VipinYalcinkaya, FatmaKumar, ManishYamabuki, KazuhiroKumar, PradeepYamaguchi, MasahikoKumar, BrajeshYamamoto, TakaoKumar, SubhalakshmiYamamoto, TakashiKumar Mishra, YogendraYamato, MasafumiKumeria, TusharYan, DayunKunioka, MasaoYan, LiangKuo, Tsung-RongYan, JipengKuo, Chung-WenYan, LiboKupka, TeobaldYang, HuaiKurc, BeataYang, Teng-ChunKurcok, PiotrYang, JingKuroda, KosukeYang, Jye-ShaneKusano, ShuheiYang, WeiKusano, YukihiroYang, Chen-IKuzuya, AkinoriYang, ShaoweiKwiecień, IwonaYang, ChengKwon, Tae-YubYang, XuanKwon, Young-WanYang, Jen-ChangKwon, Oh SeokYang, HsiharngKyle, StuartYang, EuntaeKyritsis, AportolosYang, NaitaoKyzioł, AgnieszkaYang, ZhaogangLa Mantia, Francesco PaoloYannakopoulou, KonstantinaLa Mendola, DiegoYao, Zi-JianLaaksonen, PäiviYao, JianŁabanowska, MariaYarlagadda, VenkataLabet, MarianneYates, Matthew Z.Labidi, JalelYe, LongLachman-Senesh, NoaYe, QunLadavos, Athanasios K.Ye, ZhibinLade, HarshadYen, Ming-ShienLadero Galán, MiguelYen, Hung-JuLai, YuekunYeo, HyeonukLai, Jui-YangYeom, JunseokLallana, EnriqueYi, Dong KeeLamberti, LucianoYing, HanzeLamprou, DimitriosYoo, Dong JinLanceros, SenentxuYoo, Chang GeunLang, PeterYoon, HyeonseokLange, HeikoYoon, MinyoungLanger, Jerzy JYoshida, EriLanzi, MassimilianoYoshihara, HiroshiLapitsky, YakovYoshikawa, KenichiLaPointe, AnneYoshitake, HideakiLappan, UweYou, JungmokLara-Sanchez, AgustinYounes, HusamLarraneta Landa, EnekoYousefi, Azizeh-MitraLarreta-Garde, VéroniqueYu, HaifengLaschewsy, AndreYu, Tzyy LeonŁatka, LeszekYu, JiayiLattuada, MarcoYu, Deng-GuangLaura, MorettiYu, JianLavina, SandraYu, JaecheulLavorgna, MarinoYu, LuLayek, BuddhadevYuan, JunqiLazzara, GiuseppeYuan, LeiLe Moigne, Nicolasyuan, LiLe Ouay, BenjaminYuan, HuipinLean, Meng H.Yuan, JinkaiLeBlanc, RogerYusa, Shin-ichiLecomte, PhilippeZabihi, OmidLedeţ, IonuţZaccone, AlessioLedo, AnaZafar, MuhammadLedwon, PrzemyslawZajickova, ZuzanaLee, JunghoonŻamojć, KrzysztofLee, Yong-kyuZanchetta, ErikaLee, Young-ChulZannotti, MarcoLee, SzetsenZaquen, NeomyLee, JaehwiZaręba, Jan K.Lee, Li-TingZarejousheghani, MashaalahLee, Jin-KyunŻarnowiec, WiolettaLee, WonohZarrelli, MauroLee, KangwonZarzar, Lauren D.Lee, Bang YeonZavarise, GiorgioLee, Young-ilZechel, StefanLee, BunyeoulZeng, YiningLee, Jun SeokZeng, ShuwenLee, Chul-HeeZeng, HuaqiangLee, Shyi-LongZeng, Minxiang (Glenn)Leem, Jung WooZha, ShangwenLeffler, HakonZhang, XiangLeger, LilianeZhang, WujieLéger, BastienZhang, ZhenhuaLegutko, StanislawZhang, YingyueLehocký, MarianZhang, WeiLemma, SolomonZhang, RunanLen, ChristopheZhang, XiaoxinLeporatti, StefanoZhang, BaoxinLesiak, PiotrZhang, YuLesiuk, GrzegorzZhang, QichunLevin, DavidZhang, QianqianLevy-Ontman, OshratZhang, ChunfuLi, QiangZhang, ZhienLi, KaichangZhang, ChaoqunLi, BangjingZhang, ZhiyueLi, JunshengZhang, XuefengLi, YiwenZhang, XinyuanLi, ShaojunZhang, MengLi, MeichunZhang, XiaxingLi, ChuanZhang, HuiliangLi, LongyuZhang, XuanjunLi, ZibiaoZhang, AfangLi, YuzhanZhang, YiLi, JunrongZhang, Xueqiang AlexLi, PingZhang, HaiLi, XindaZhang, YongLi, RuosongZhang, YanlinLi, JianyuZhang, SuiLi, YingZhang, XiaoxingLi, YanZhang, Da-MingLi, ZhaohuiZhao, LianfengLi, ShanghaoZhao, YueLi, JinhongZhao, ZongminLi, ZhenZhao, WeifengLi, JingZhao, LingLi, YunchaoZhao, ZhenghangLi, HongxiaZhao, JunpengLi, JiantongZhao, FeipingLi, ZhiZhao, NingningLi, QianZhao, Shu-NaLi, JinghaoZhao, XinxinLi, AnlongZheng, FanLiang, WenyanZheng, ZhaoliangLiang, KangZheng, QingbinLiao, Shu-ChuanZheng, BotongLiaw, Chya-YanZheng, DafengLichtenhan, Joseph D.Zhi, FangLiebscher, MarcoZhong, TuhuaLienkamp, KarenZhong, LinxinLignola, Gian PieroZhou, YiqunLikodimos, VlassisZhou, YonghuiLikozar, BlažZhou, ShengtaiLim, KhoonZhou, KunLim, Eun-KyungZhou, ZuowanLin, XiaoshanZhou, XingpingLin, Chia-HerZhou, ZhengpingLin, Ching HsuanZhou, DezhongLin, Hung-YinZhou, ShuangLin, RijiaZhou, YouLin, XiaojieZhou, ZhuxianLin, Gwo-GengZhu, JieLin, Yang-weiZhu, Yingxi ElaineLin, HaijingZhu, FulongLin, Ching-HsuanZhu, XiangweiLin, Cheng-WeiZhu, GuangshanLin, Chao-SungZhu, WeiLing, JunZhu, MeifangLinul, EmanoilZiąbka, MagdalenaLionetto, FrancescaZiats, Nicholas P.Liou, Guey-ShengŽic, MarkLiparoti, SaraZimoch-Korzycka, AnnaLiqun, ZhangZitzenbacher, GernotLittle, MarcZolfagharian, AliLitvinov, VictorZoltán, PásztiLiu, FeiZsuga, MiklósLiu, JinglinZukowski, WitoldLiu, JinyunZuo, JianLiu, Xian HuZyła, GawełLiu, Song


